# Professional Mechanical Tooth Cleaning Method for Dental Implant Surface by Agar Particle Blasting

**DOI:** 10.3390/ma14226805

**Published:** 2021-11-11

**Authors:** Hideaki Sato, Hiroshi Ishihata, Yutaka Kameyama, Ryokichi Shimpo, Satoshi Komasa

**Affiliations:** 1 Department of Mechanical Engineering, Faculty of Science and Engineering, Tokyo City University, 1-28-1 Tamadutsumi, Setagaya-ku, Tokyo 158-8557, Japan; shsato@tcu.ac.jp (H.S.); ykameya@tcu.ac.jp (Y.K.); rshimpo@tcu.ac.jp (R.S.); 2 Department of Periodontology and Endodontology, Graduate School of Dentistry, Tohoku University, 4-1 Seiryo-machi, Aoba-ku, Sendai 980-8575, Japan; hiroshi.ishihata.a8@tohoku.ac.jp; 3 Department of Removable Prosthodontics and Occlusion, Osaka Dental University, 8-1 Kuzuha-hanazono-cho, Hirakata-shi, Osaka 573-1121, Japan

**Keywords:** oral implants, peri-implantitis, abutment, professional mechanical tooth cleaning, agar particle, blasting, surface roughness

## Abstract

Oral dysfunction due to peri-implantitis and shortened life of implants has become a major concern. Self-care and removal of oral biofilms by professional mechanical tooth cleaning (PMTC) are indispensable for its prevention. However, if the surface roughness of the implant is increased, it may result in the adhesion of biofilm in the oral cavity. Therefore, the PMTC method can serve for long-term implant management. Calcium carbonate (CaCO_3_) has been used as a cleaning method for implant surfaces; however, there is concern that the implant surface roughness could increase due to particle collision. Therefore, in this study, to establish a blasting cleaning method that does not adversely affect the implant surface, a new blasting cleaning method using agar particles was devised and its practical application examined. When the simulated stains were blasted with white alumina (WA) abrasive grains and CaCO_3_ particles, the simulated stains were almost removed, the surface roughness changed to a satin-finished surface—which was thought to be due to fine scratches—and the surface roughness increased. Most of the simulated stains were removed on the surface of the sample blasted with glycine particles and agar particles. Conversely, the gloss of the sample surface was maintained after cleaning, and the increase in surface roughness was slight.

## 1. Introduction

Implant treatment is an indispensable option for the prosthetic treatment of tooth defects, and patients’ need for this treatment is very high [1,2]. Pure titanium and titanium alloys are the main implant materials used. Titanium surfaces are biocompatible and have excellent mechanical properties, and when implanted in a living body, cause osseointegration with bone, making it useful as a biomaterial. Unlike natural peri-dental soft tissue, the peri-implant soft tissue does not have a sealing structure and is fragile. Therefore, the mucosa around the implant has a high risk of infection. If inflammation occurs around the implant, the spread of inflammation is much faster than with natural teeth, and the risk of peri-implantitis and the implant itself falling off is very high [3,4,5].

Despite the progress of implant treatment, peri-implantitis is becoming an increasingly frequent concern in dental clinics. Peri-implantitis is an inflammatory lesion caused by periodontal disease bacteria that infect the area around the implant [6,7,8]. Chronic infections, similar to chronic periodontitis, are characterized by redness, swelling of the peri-implant mucosa, and destruction of the peri-implant bone [9,10,11]. Bacteria that are frequently detected in chronic periodontitis activity are detected in the implant body that has developed peri-implantitis. The presence of oral bacteria during implant placement affects biofilm formation on the surface of the implant body. Therefore, the biofilm present in the subgingival plaque and periodontal pockets of the remaining teeth can be a medium of bacteria that colonize the body of the newly placed implant [12,13,14,15].

Currently, the main techniques used to treat peri-implantitis include chemotherapy. Initial lesions can be resolved by mechanical cleaning of the implant body [16,17,18,19]. Chemotherapy for the treatment of peri-implantitis can cause side effects [20,21]. Currently, there is no cure for peri-implantitis. Although each treatment has its risks, treatment of peri-implantitis is defined as the removal of tartar and plaque on the surface of the implant body. Methods for cleaning the contaminated implant body include mechanical cleaning with a scaler, chemical cleaning with phosphoric acid, laser, and plasma treatment [22,23]. Unlike natural teeth, the hardness of the implant surface is soft, so the quality of cleaning varies depending on the skill of the doctor. Therefore, in patient management to achieve long-term use of implants, it is necessary to establish effective professional mechanical tooth cleaning (hereinafter referred to as PMTC) that can prevent the onset of peri-implantitis.

This study proposes a blasting cleaning method for dentists and dental hygienists to perform PMTC regardless of their skill differences [24,25,26]. Currently, a method for removing plaque by blasting of tricalcium phosphate (β-TCP) particles onto plaque containing periodontal pathogenic bacteria adhering to the surface of the abutment is being studied [27,28,29,30,31]. However, there is concern that the surface roughness of the abutment can increase owing to particle collisions. Increased surface roughness of the abutment increases the adhesion of biofilm and exacerbates peri-implantitis [32,33,34]. Therefore, the focus of this study is on soft and gentle agar particles in living organisms.

The purpose of this study is to analyze the effects of various particles, such as agar particles, on the surface of implant materials. The development of a new PMTC method that can be easily performed without damaging the implant surface, regardless of the skill of the dentist, can be expected.

## 2. Materials and Methods

### 2.1. Effect of Difference in Jet Particles on Titanium Surface

#### 2.1.1. Sample Preparation

Pure titanium test pieces were machined from 6 mm thickness JIS Type 2 pure titanium sheets. A rectangular test piece was ground with dimensions of 10 × 15 mm using a surface grinding machine and diamond wheel (SD140, Asahi Diamond Industrial Co., Ltd., Tokyo, Japan). In addition, the initial arithmetic mean surface roughness, Ra, of the test piece before blasting was adjusted and polished so that Ra ≈ 0.05 µm.

#### 2.1.2. Injection Device and Injection Particles

**Figure 1:**
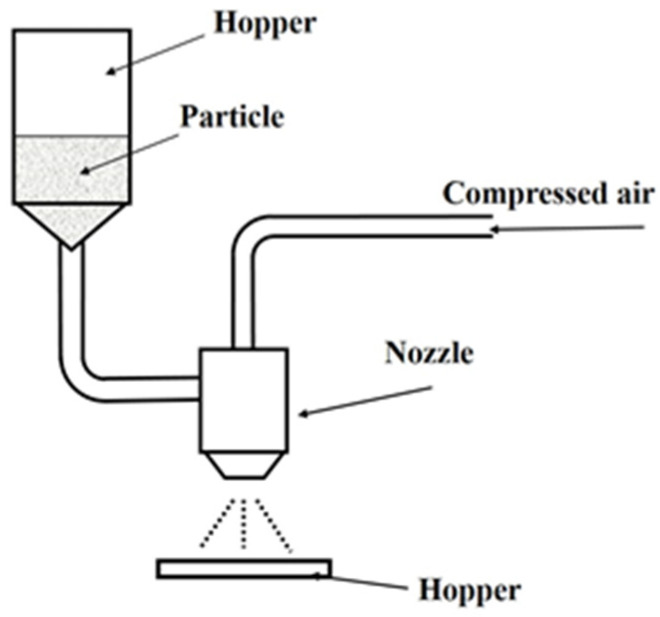
Blasting equipment.

**Figure 2:**
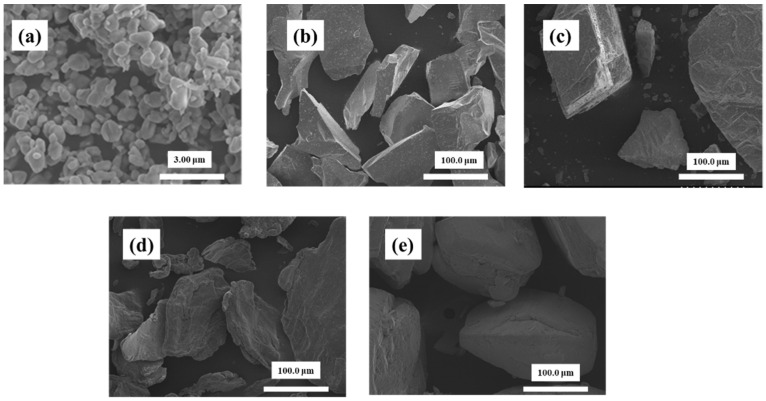
SEM images of particles. The particles used in this study were: β-TCP particles (**a**), white alumina particles (**b**), calcium carbonate particles (**c**), agar particles (**d**), and glycine particles (**e**).

Figure 1 shows the outline of the blasting equipment. The blasting equipment is a device for blasting particles onto a test piece using compressed air. It uses the same suction method as the clinical device. The particles used in this study were: β-TCP particles (Taihei Kagaku Sangyo Co., Ltd., Osaka, Japan), white alumina particles (WA particles, Fuji Seisakusho Co., Ltd., Tokyo, Japan), calcium carbonate particles (CaCO_3_ particles, Maruo Calcium Co., Ltd., Akashi, Japan), agar particles (Ina agar type S-6, Ina Food Industry Co., Ltd., Nagano, Japan), and glycine particles (Organic Synthetic Chemicals Industry Co., Ltd., Tokyo, Japan) (Figure 2).

#### 2.1.3. Experimental Methods

The experimental evaluation used arithmetic mean surface roughness Ra, maximum height roughness Rz, glossiness, and surface observation. The target roughness was the arithmetic mean surface roughness Ra = 0.2 µm and Rz = 1.8 µm or less. According to Rimondini et al. [35], when the arithmetic mean surface roughness Ra of the titanium surface was 0.21 µm or less and the maximum height roughness Rz was 1.780 µm or less, the plaque adhesion to the titanium surface in the oral cavity was suppressed. A stylus-type surface roughness meter (Mitutoyo Co., Ltd., Kawasaki, Japan, SURFTEST SJ-400, and Tokyo Seimitsu Co., Ltd., Tokyo, Japan, Surfcom FLEX-50A) was used to measure the surface roughness and cross-sectional curve. The arithmetic mean surface roughness and maximum height roughness were measured ten times for each test piece, and the average value was calculated. Glossiness was measured using a glossiness meter (HORIBA, Ltd., Kyoto, Japan, high-gloss checker IG-410, Gs (60°)). Glossiness measurement is a newly devised measurement for evaluating the esthetics of titanium surfaces. An optical microscope (KEYENCE CORPORATION, Osaka, Japan, VHX-700F) was used for surface observations. After obtaining the initial surface roughness of the machined flat surface test piece of pure titanium, the surface roughness and glossiness were measured, and the surface was observed. Then, an oil-based pen was applied to the titanium surface as a simulated stain. Each particle was blasted (blasting pressure 0.2 MPa, distance to the sample, 20 mm) into the test piece while changing the injection time (5, 10, and 30 s). After blasting, the test piece was washed with tap water to remove particles adhering to the surface, and surface roughness measurements, glossiness measurements, and surface observations were performed.

### 2.2. Effect of Different Agar Particles on the Titanium Surface

#### 2.2.1. Effect of Different Sizes of Agar Particles on the Dirt on the Titanium Surface

**Figure 3:**
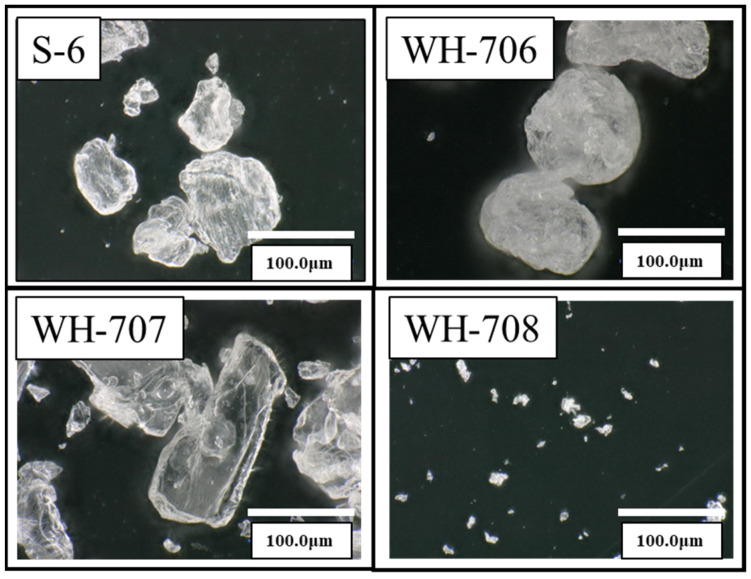
SEM images of agar particle are shown. S-6 is powdery and has an average diameter of 102 µm, WH-706 is spherical and has an average diameter of 87 µm, WH-707 is scaly and has an average diameter of 105 µm, and WH-708 is a fine powder and has an average diameter of 8 µm.

Based on the results of the first experiment, agar blasting was performed using five types of particles (Figure 3), where S-6 was powdery and had an average diameter of 102 μm, WH-706 was spherical with an average diameter of 87 μm, WH-707 was scaly and had an average diameter of 105 μm, and WH-708 was a fine powder with an average diameter of 8 μm. For the test piece, titanium was used as a model for the implant abutment. The surface roughness Ra was adjusted to 0.09 μm ≤ Ra ≤ 0.10 μm. An oil-based marker (Zebra Co., Ltd., Tokyo, Japan, Hi-McKee MO-150-MC) was used as the simulated plaque stain. Oil-based markers are widely used in dentistry.

Using a diamond wheel (SDC140, Asahi Diamond Industrial Co., Ltd., Tokyo, Japan) and a surface grinding machine, the pure titanium test piece was machined into a rectangular test piece of about 15.0 mm × 10.0 mm × 6 mm, and the surface of about 15.0 mm × 10.0 mm was used as the test surface. The arithmetic average roughness Ra of the test piece was adjusted to 0.09 μm ≤ Ra ≤ 0.10 μm using # 240 to # 3000 water-resistant abrasive paper (FUJISTAR: manufactured by Sankyo Rikagaku Co., Ltd., Saitama, Japan). Subsequently, agar particles were blasted onto the test surface using the blasting equipment while keeping the blasting conditions constant. After the blasting, surface roughness and glossiness were measured, and the surface of the test piece was observed using an optical microscope.

#### 2.2.2. Effect of Different Sizes of Agar Particles on the Tartar Model on the Titanium Surface

Tartar was examined as a factor involved in peri-implantitis. For the calculus model, which is a calculus, calcium carbonate was attached to the surface of the test piece using the underwater thermal substrate method, as described by Takagi et al. [36]. Contrex (Contrex^®^ Nestlé, Vevey, Switzerland) was placed in an electric kettle (Tiger Corporation, Tokyo, Japan, PDR-G221W) and heated to about 90 °C. Next, titanium was connected to an electric circuit where the voltage was 5.0 V and the current was 0.40 A. The energized titanium was immersed in heated Contrex for approximately 9 h to deposit a calcified film on the surface of the test piece. Agar particles were injected into the tartar model under blasting conditions. Cleaning by the blasting of each agar particle was evaluated by observing the surface of the test piece before and after blasting.

### 2.3. Statistical Analysis

Each measurement was performed three times and statistical analyses were performed by one-way analysis of variance. When a significant difference was found, the Mann–Whitney U Test was used. The significance level was <5%.

## 3. Results

### 3.1. Effect of Difference in Jet Particles on Titanium Surface

**Figure 4:**
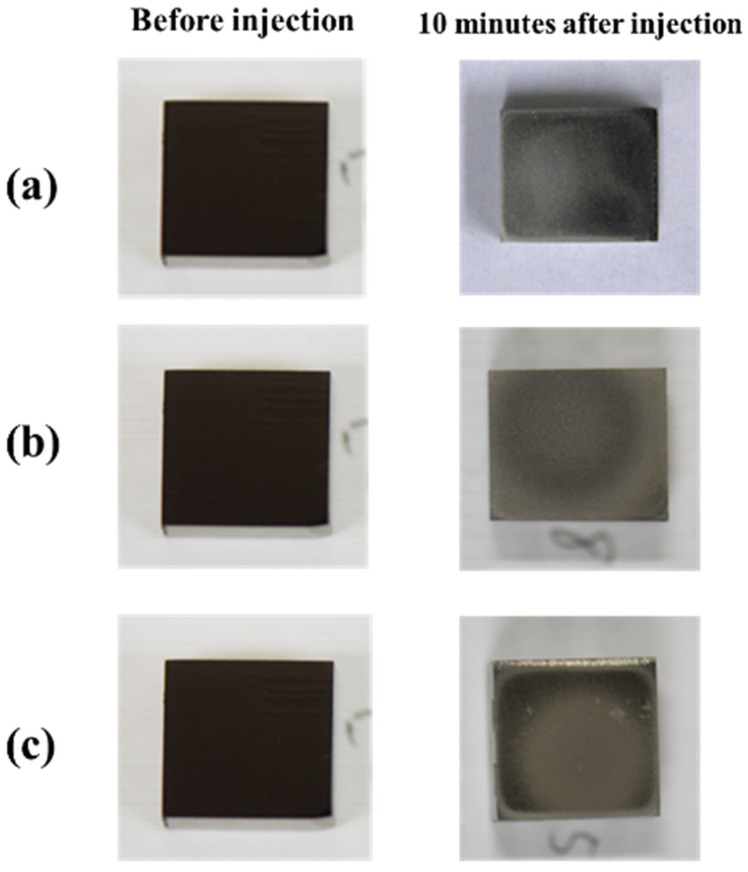
Images of the titanium surface before and after 10 s blasting of β-TCP (**a**), CaCO_3_ particles (**b**), and white alumina particles (**c**).

**Figure 5:**
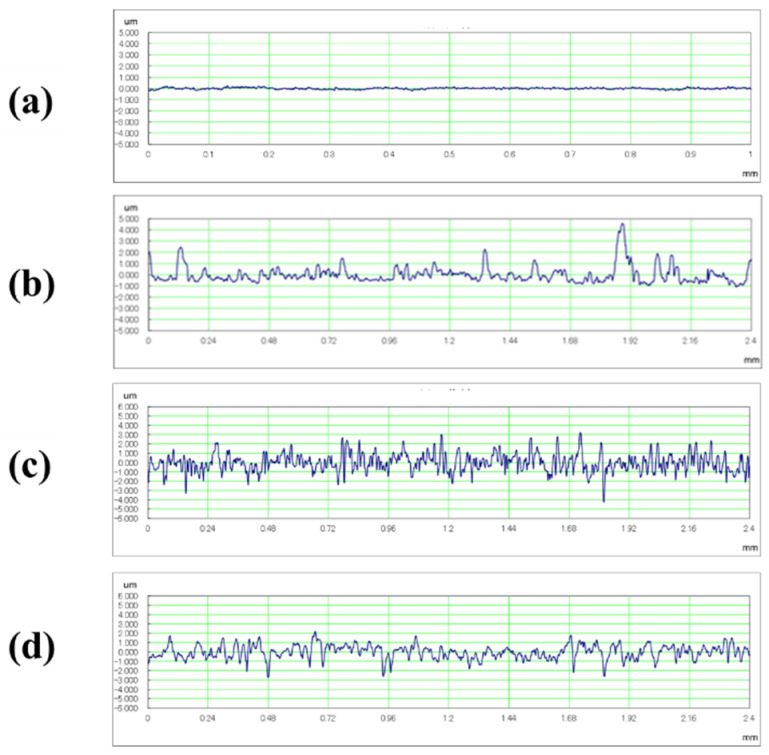
The cross-sectional curves before blasting and after 10 s blasting of each particle are shown: (**a**) before blasting, (**b**) after blasting of β-TCP, (**c**) after blasting of WA particle, (**d**) after blasting of CaCO_3_ particle. It can be confirmed that the surface of all particles was greatly roughened.

**Figure 6:**
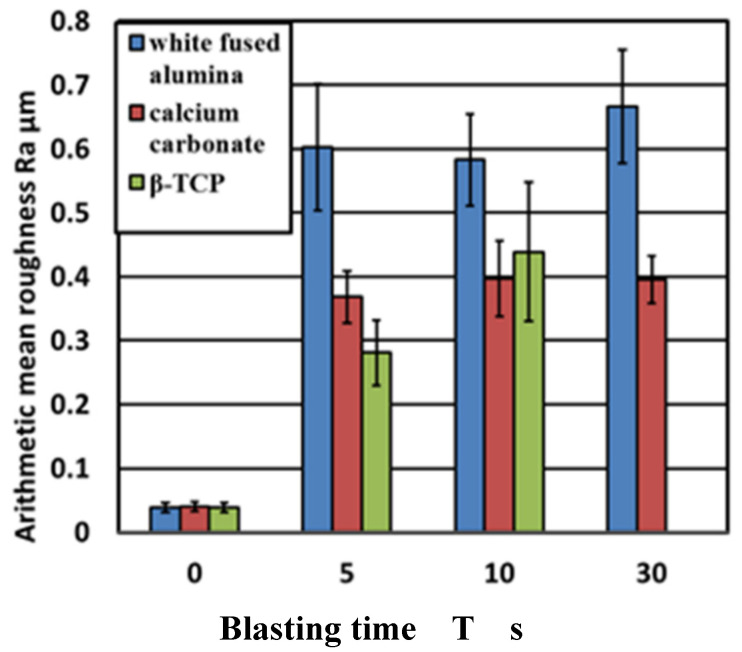
The changes in surface roughness are shown. It was confirmed that the surface roughness increased significantly under all conditions. From this, the target roughness Ra = 0.2 µm was greatly exceeded, and there is concern about plaque adhesion.

**Figure 7:**
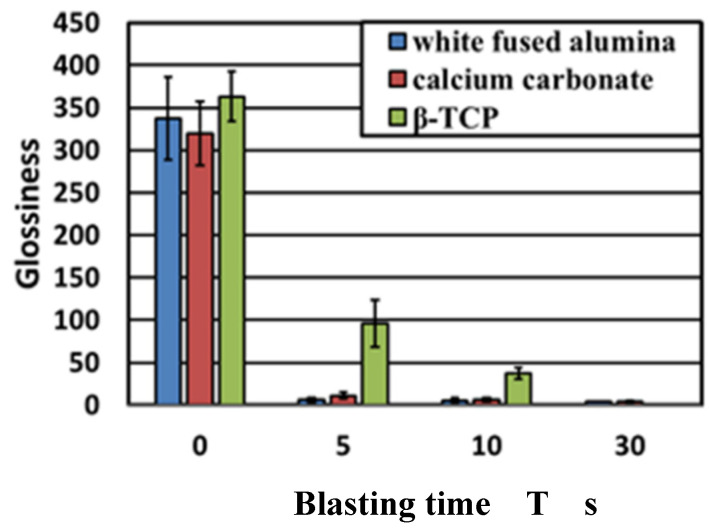
The changes in glossiness are shown. In the blasting of WA particles and CaCO_3_ particles, the glossiness shows almost zero. Although β-TCP has a slight decrease in glossiness compared with other particles, it has a significant decrease. Considering that a part of the particles has changed to a satin-finished surface, it affects the aesthetics.

Images of the titanium surface before and after 10 s blasting of WA particles, CaCO_3_ particles, and β-TCP, are shown in Figure 4. It was noted that the ink was cleaned when all the particles were blasted, however, the entire test surface later changed to roughed surface upon blasting with WA and CaCO_3_ particles, resulting in a loss of glossiness. In the β-TCP injection, it was confirmed that part of the test surface changed to a satin-finished surface, and the surface changed. The cross-sectional curves before blasting and after 10 s of blasting of each particle are shown in Figure 5. It was confirmed that the surfaces of all particles were greatly roughened. Figure 6 shows the change in surface roughness. It was confirmed that the surface roughness increased significantly under all conditions. From this, the target roughness Ra = 0.2 µm was greatly exceeded, yielding to the concern about plaque adhesion. Figure 7 shows the changes in the glossiness. In the blasting of WA and CaCO_3_ particles, the glossiness was almost zero. Although β-TCP had a small decrease in glossiness compared with other particles, it had a large decrease considering that some of the particles changed to a satin-finished surface, which affected the esthetics. 

It was confirmed that the ink was cleaned when all the particles were blasted. However, it was confirmed that the entire test surface was changed to a satin-finished surface by the blasting of WA particles and CaCO_3_ particles, and the gloss was lost. In the β-TCP injection, it was confirmed that a part of the test surface had changed to a satin-finished surface, and the surface changed.

**Figure 8:**
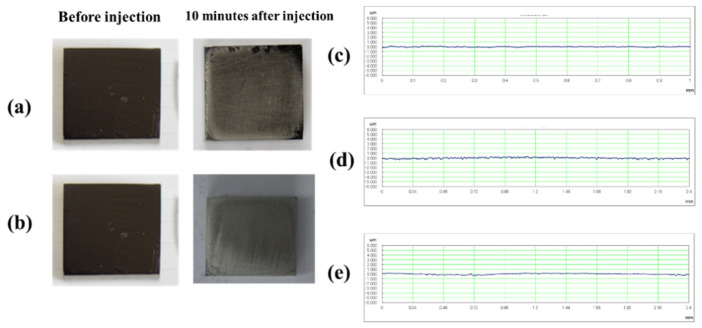
Images of the titanium surface before and after 10 s blasting of: (**a**) agar, and (**b**) glycine particles, are shown. The cross-sectional curves before blasting and after 10 s blasting of each particle are shown: (**c**) before blasting, (**d**) after blasting of agar, (**e**) after blasting of glycine particle.

**Figure 9:**
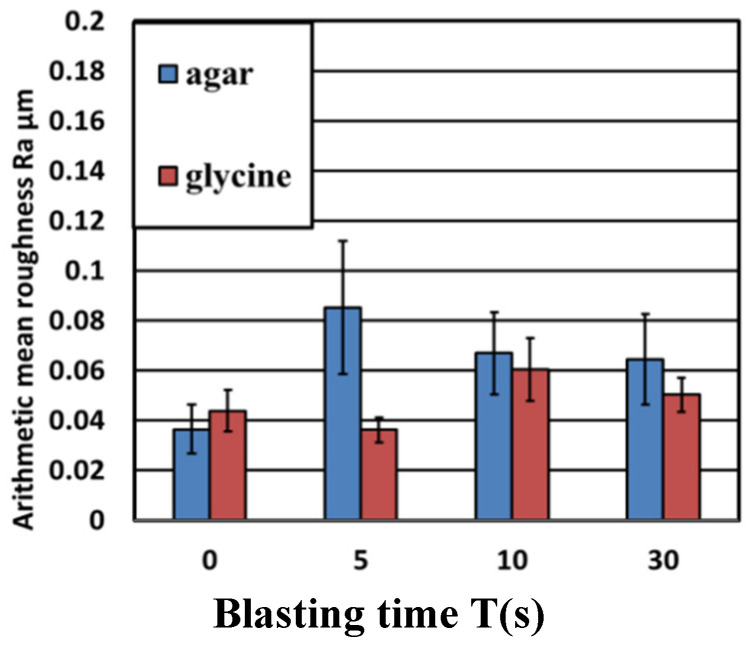
The changes in surface roughness are shown. The increase in surface roughness is slight and well below the target roughness Ra = 0.2 µm, so even if agar particles and glycine particles are blasted, the effect on the surface texture of the abutment is very small.

**Figure 10:**
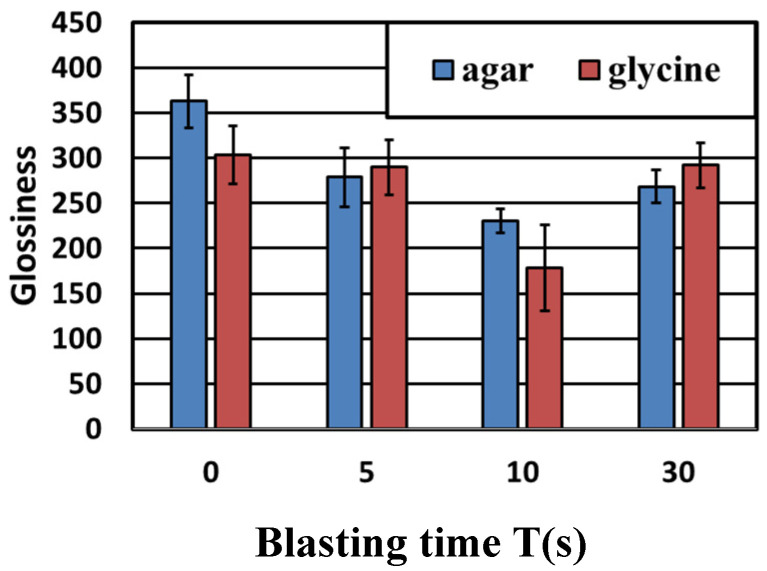
The changes in glossiness are shown. After blasting, glossiness is reduced, but the rate of reduction is low. Gloss remains on the blasted surface, and changes cannot be visually confirmed.

Figure 8 shows images of the titanium surface before and after the blasting of agar and glycine particles. It was confirmed that the ink was cleaned when the agar particles and glycine particles were blasted. In the blasting of agar particles, ink remained on the edge of the test piece. However, the areas where the particles impacted were sufficiently cleaned; this constituted no major issue. In addition, the test surface remained glossy and had only a little effect on esthetics. Figure 8 shows the cross-sectional curves. There was almost no change in the surface area before and after blasting. Figure 9 shows the change in surface roughness. Figure 9 shows that the increase in surface roughness was slight and well below the target roughness Ra = 0.2 µm, so even if agar particles and glycine particles were blasted, the effect on the surface texture of the abutment would be a little. Figure 10 shows the changes in glossiness. After blasting, glossiness was reduced to a small extent. As shown in Figure 8, gloss remained on the injection surface, and the changes were merely visible. Therefore, it was considered that agar particles’ blasting has almost no effect on esthetics.

### 3.2. Effect of Different Sizes of Agar Particles on the Dirt on the Titanium Surface

**Figure 11:**
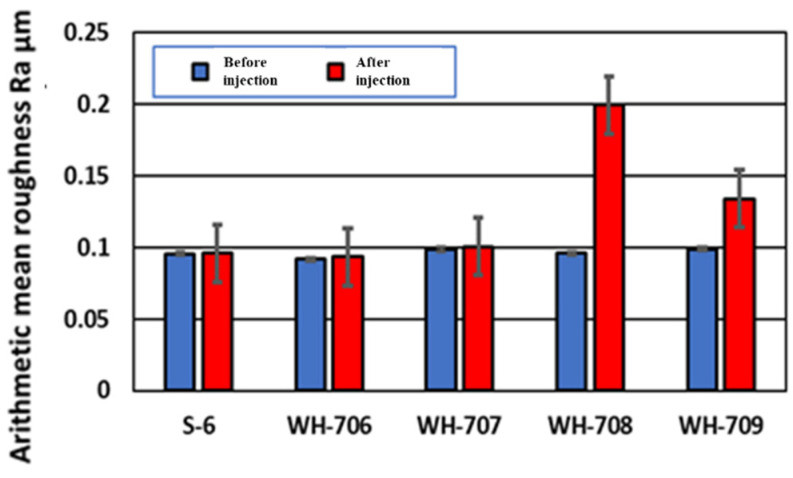
The arithmetic mean roughness Ra of the test piece surface for each simulated stain is shown.

**Figure 12:**
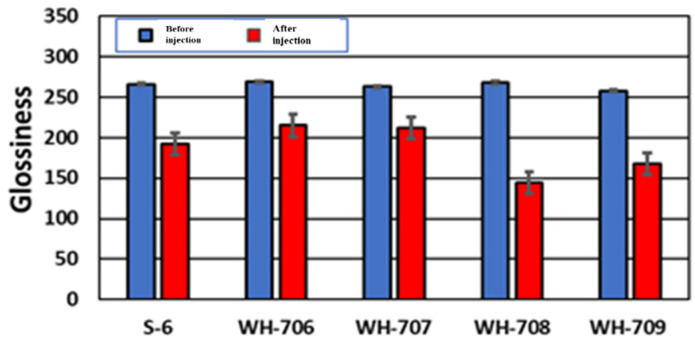
The glossiness of the test piece surface for each simulated stain is shown.

**Figure 13:**
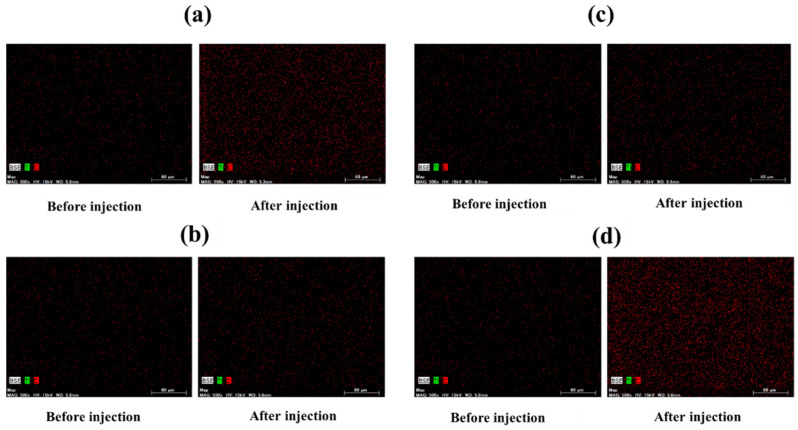
Elemental analysis images before and after agar particle blasting are shown: (**a**) S-6, (**b**); WH-706, (**c**); WH-708, (**d**); WH-709 polishing compared with before adhesion. Compared with the other four types of agar, the amount of carbon after blasting was the lowest.

Figure 11 and Figure 12 show the arithmetic mean surface roughness Ra and the glossiness of the test piece surface for each simulated stain, respectively. When a small size agar comes into contact with air, the particles stick to each other and tend to form small aggregates such as WH-708 and WH-709. Therefore, it was found that the surface roughness of the test piece was large, and the glossiness was lowered; thus, it was not suitable for cleaning. As shown in Figure 13, red indicates carbon. A very small amount of carbon was confirmed on the surface after polishing and before adhesion. After blasting WH-706, some carbon remained after polishing compared with before adhesion. Compared with the other four types of agar, the amount of carbon after blasting was the lowest. The black part is a color unrelated to the element and is an image displayed only with carbon.

### 3.3. Effect of Different Sizes of Agar Particles on the Tartar Model on the Titanium Surface

**Figure 14:**
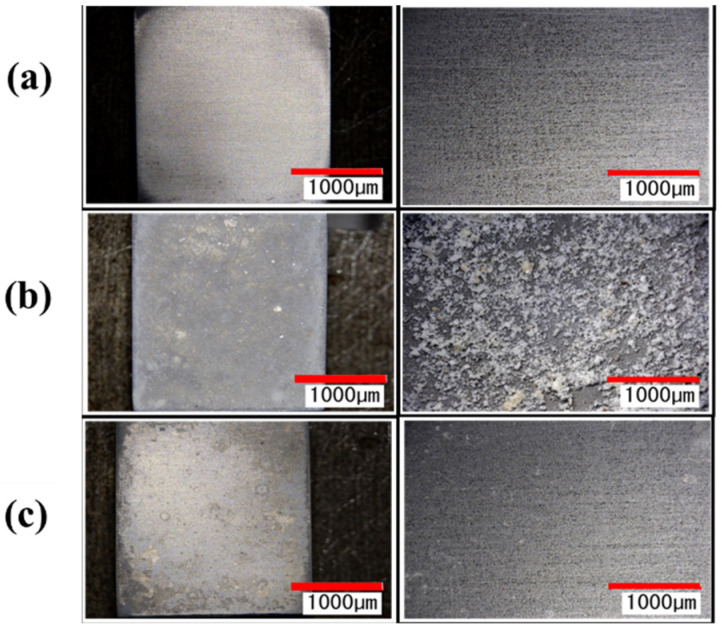
The images of the surface of the test piece before and after the deposition of the tartar model and before and after the blasting of the agar particles, are shown. (**a**) is the sample surface (polished surface) before the tartar model was deposited, (**b**) is the sample surface of tartar model, and (**c**) is the sample surface after the blasting by WH-706.

**Figure 15:**
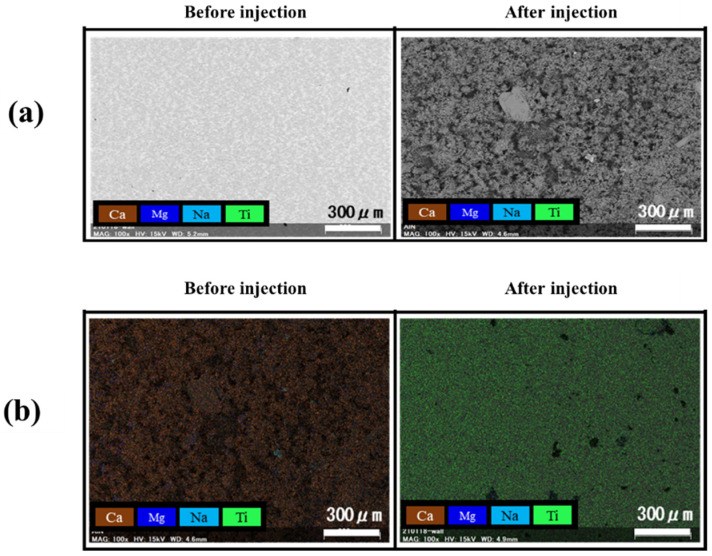
(**a**) is an SEM image before and after the tartar model deposition, (**b**) shows SEM images after deposition (tartar model) and after blasting with agar particles by WH-706. Brown in the figure represents Ca, blue represents Mg, light blue represents Na, and green represents Ti.

Figure 14 shows the surface of the test piece before and after the formation of the tartar model, and before and after the blasting of 25 g agar particles. Figure 15 shows a tabletop scanning electron microscope (SEM) image of the surface of the test piece before and after the formation of the tartar model and the surface analysis image. From Figure 14 and Figure 15, it was confirmed that calcium carbonate was attached by the underwater thermal substrate method. In addition, from Figure 14, it was confirmed visually that calculus, which is a tartar model, was removed. Figure 15 reveals that Ca shown in brown before blasting was not seen after blasting, but Ti was seen in the entire figure after blasting; thus, the removal of the tartar model could be confirmed by elemental analysis [34]. As shown in Figure 15, the Ra after blasting in the blasting of particle WH-706 was slightly smaller than that before injection. Ra increased when other particles were blasted.

## 4. Discussion

Peri-implantitis is a reversible inflammation localized to the soft tissue around the implant. This is thought to be due to biofilm present in the tissue surrounding the implant and has symptoms similar to those of the original periodontitis [1,2,3,4,5,6,7,8,9,10]. However, peri-implantitis requires appropriate treatment because of the rapid and widespread infiltration of inflammatory cells compared with periodontitis. Peri-implantitis is caused by a bacterial infection associated with biofilm adhesion. Therefore, it is important to remove biofilm on the surface of the implant material during the treatment of peri-implantitis. This study aimed to develop a PMTC method for treating peri-implantitis by applying various particles using blasting equipment, regardless of the skill of dentists and dental hygienists. Although simulated dirt removal was achieved in all particle groups and the effect of blasting was confirmed, the surface roughness of the test pieces in the WA and CaCO_3_ groups increased. These particles may increase the surface roughness of the implant body. On the other hand, in the glycine and agar groups, the increase in surface roughness was small, and the effect on the surface texture was considered to be small. Moreover, when the shapes of the agar particles were compared, the cleaning results of WH-706, which had an almost spherical shape, showed the best results with the agar particles. Furthermore, it was clarified that agar blasting was also useful for the tartar model created on the titanium surface.

Bacterial adhesion is observed when the surface of the implant body falls off, for a patient with peri-implantitis [32,33,34]. It has been reported that titanium surfaces are the most prone to biofilm adhesion, although they depend on the metal material used in the dental prosthesis [37,38,39]. Numerous reports have shown that roughened pure titanium implant materials induce early adhesion of bone marrow cells and achieve early osseointegration [40,41,42]. Notwithstanding, it has been clarified that titanium implants becomes a hotbed for bacterial adhesion; thus, it is essential to develop a cleaning system that removes the biofilm on the implant surface without damaging the implant surface [43]. Air-abrasives using each particle are attracting attention as a method of periodontal treatment and professional care for patients with peri-implantitis [44]. β-TCP particles that have been studied using the PMTC method [45] have a Vickers hardness of HV 400 and a density of 3.14 g/cm^3^. WA particles have a proven track record of being used as a blasting material for caries treatment with a Vickers hardness of HV 2000 and a density of 4 g/cm^3^. CaCO_3_ particles are used as blasting particles for cleaning the tooth surface, are contained in some commercially available toothpastes, and are widely used in the dental field. It has a Vickers hardness of HV 300 and a density of 2.71 g/cm^3^. It was confirmed that the ink was cleaned when all the particles were blasted. However, the entire test surface was changed to a satin-finished surface by the blasting of WA and CaCO_3_ particles, and the gloss was lost. In the β-TCP blasting, it was confirmed that a part of the test piece surface changed to a satin-finished surface, and the surface roughness changed. It was confirmed that the surface roughness increased significantly under all the conditions. 

Thus, the target surface roughness Ra = 0.2 µm was greatly exceeded, and plaque adhesion was a concern. From this, it was confirmed that the surface of the pure titanium abutment was scratched after blasting particles. In the blasting of WA and CaCO_3_ particles, the glossiness was almost zero, and it was confirmed that the esthetics of the test surface was greatly reduced considering that the test surface changed to a satin-finished surface. Although the decrease in the glossiness of β-TCP was small compared with other particles, it was significantly reduced, and it was considered that β-TCP affects the esthetics, considering that some of the particles have changed to a satin-finished surface. From the above results, it was found that the surface roughness was greatly increased when conventional particles were blasted, which also affected the esthetics. It was confirmed that the ink was cleaned when the agar particles and glycine particles were blasted. In the blasting of agar particles, ink remained on the edge of the test piece. However, the areas where the particles impacted were sufficiently cleaned; this was not a major problem. In addition, the test piece surface remained glossy, and the effect on the esthetics was almost negligible. As the increase in surface roughness was small and well below the target roughness Ra = 0.2 µm, it was considered that the effect of blasting agar particles and glycine particles on the surface texture of the abutment was negligible. After blasting, the glossiness was reduced, but the rate of decrease was low. The blasted surface remained glossy, and the changes were merely visible. Therefore, agar blasting almost did not affect the esthetics. Glycine is used as a food additive, has high biocompatibility, and is safe. It has been reported that the cleaning surface is highly smooth, but the cleaning power is weak; therefore, the focus of this work was on agar particles.

Agar with a small particle size of WH-708 and WH-709 tended to form small aggregates because the particles could stick to each other when exposed to air. Therefore, it was found that the surface roughness of the test piece was increased, and the glossiness was decreased; thus, it was not suitable for cleaning. When the results of the elemental analysis were considered, the surface of JIS Type 2 pure titanium after polishing with water-resistant abrasive paper contained a large amount of titanium (green), and almost no carbon could be confirmed. After that, when an oil-based marker was attached, a large amount of carbon (red) contained in the oil-based marker was observed. A very small amount of carbon was confirmed on the surface after polishing and before adhesion; on the surface after blasting WH-706, more carbon was removed than before adhesion. Compared with the other four types of agar, the amount of carbon after blasting was the lowest. It was also considered that tartar could be removed by agar blasting into the abutment cleaning. Among them, it was found that cleaning results with the agar particles WH-706 were particularly excellent. This was because the shape of agar particles are almost spherical, and it seems that the shape is suitable for cleaning. Thus with the spherical shape, when agar particles were impacted with the tartar model by blasting, a uniform force could be applied regardless of the direction of the particles’ motion, so more tartar could be removed. With non-spherical particles, the force applied to the tartar model becomes non-uniform in the direction of the particles that come into contact with it, leading to an increase in surface roughness. Therefore, spherical agar particles were considered to be the best shape for cleaning. Agar is a biocompatible food and is a soft particle that does not easily damage the abutment. In addition, because of its low cost, it is expected to be used as a PMTC method for peri-implantitis.

Compared with other particles, it was confirmed that agar particles blasting could remove stain and tartar. In particular, it was confirmed that when the shape of the agar particles was spherical, the change in surface roughness was small, and in addition to being able to hold implants that are difficult for plaque to adhere to, it was possible to remove more tartar. Therefore, it is believed that agar particles are effective for implant cleaning and can be applied to tooth surface cleaning and flap surgery. In the future, it is expected that agar blasting will be conducted as PMTC by verifying the cleaning effect by in vivo evaluation and downsizing the blasting equipment for clinical application.

## 5. Conclusions

The following conclusions were obtained as a result of blasting using multiple particles to develop a new PMTC that can easily be performed regardless of the skill of the dentist without damaging the implant surface. Conventionally-used blastings of WA particles, CaCO_3_ particles, and β-TCP, showed an increase in surface roughness and a decrease in glossiness, which adversely affected pure titanium, which is the material for implants. This may cause an increase in plaque adhesion to the abutment surface and is considered to be a concern as particles to be used for PMTC. In the injection of agar particles, which was devised as a new particle, no increase in surface roughness was observed, and there was a slight decrease in glossiness. Because a wide range of cleaning was possible on the rectangular test piece, cleaning can be performed regardless of the skill of the dentist. 

## Data Availability

The data presented in this study are available on request from the corresponding author.

## References

[1] Lindhe J., Karring T., Lang N.P. (2003). Clinical Periodontology and Implant Dentistry.

[2] Misch C.E. (1999). Contemporary Implant Dentistry. Implant Dent..

[3] Cairo F., Pagliaro U., Nieri M. (2008). Soft Tissue Management at Implant Sites. J. Clin. Periodontol..

[4] Tebbetts J.B., Hammond D.C. (2002). A System for Breast Implant Selection Based on Patient Tissue Characteristics and Implant-Soft Tissue Dynamics. Plast. Reconstr. Surg..

[5] Wennström J.L., Bengazi F., Lekholm U. (1994). The Influence of the Masticatory Mucosa on the Peri-Implant Soft Tissue Condition. Clin. Oral Implant. Res..

[6] Bernardi S., Bianchi S., Tomei A.R., Continenza M.A., Macchiarelli G. (2019). Microbiological and SEM-EDS evaluation of titanium surfaces exposed to periodontal gel: In vitro study. Materials.

[7] Kaiser F., Scharnweber D., Bierbaum S., Wolf-Brandstetter C. (2020). Success and side effects of different treatment options in the low current attack of bacterial biofilms on titanium implants. Bioelectrochemistry.

[8] Klinge B., Hultin M., Berglundh T. (2005). Peri-Implantitis. Dent. Clin. N. Am..

[9] Prathapachandran J., Suresh N. (2012). Management of Peri-Implantitis. Dent. Res. J..

[10] Mahato N., Wu X., Wang L. (2016). Management of Peri-Implantitis: A Systematic Review, 2010–2015. Springerplus.

[11] Mombelli A. (1997). Etiology, Diagnosis, and Treatment Considerations in Peri-Implantitis. Curr. Opin. Periodontol..

[12] Jacombs A., Tahir S., Hu H., Deva A.K., Almatroudi A., Wessels W.L.F., Bradshaw D.A., Vickery K. (2014). In Vitro and In Vivo Investigation of the Influence of Implant Surface on the Formation of Bacterial Biofilm in Mammary Implants. Plast. Reconstr. Surg..

[13] Pita P.P.C., Rodrigues J.A., Ota-Tsuzuki C., Miato T.F., Zenobio E.G., Giro G., Figueiredo L.C., Gonçalves C., Gehrke S.A., Cassoni A. (2015). Oral Streptococci Biofilm Formation on Different Implant Surface Topographies. BioMed Res. Int..

[14] Daubert D.M., Weinstein B.F. (2019). Biofilm as a Risk Factor in Implant Treatment. Periodontology.

[15] Lin H.Y., Liu Y., Wismeijer D., Crielaard W., Deng D.M. (2013). Effects of Oral Implant Surface Roughness on Bacterial Biofilm Formation and Treatment Efficacy. Int. J. Oral Maxillofac. Implant..

[16] Esposito M., Grusovin M.G., Worthington H.V. (2012). Treatment of Peri-Implantitis: What Interventions Are Effective? A Cochrane Systematic Review. Eur. J. Oral Implantol..

[17] Haritha V., Sasikumar K.G., Prathapachandran R., Anoop M. (2020). Contemplating Macrotyloma Uniflorumin Traditional Snake Envenomation Management Practices Through Analysis on Various Solvents by a Scheme of Spectroscopic, Phytochemical and Chromatographical Analysis. Int. J. Curr. Res. Biosci. Plant. Biol..

[18] Claffey N., Clarke E., Polyzois I., Renvert S. (2008). Surgical Treatment of Peri-Implantitis. J. Clin. Periodontol..

[19] Schou S., Berglundh T., Lang N.P. (2004). Surgical Treatment of Peri-Implantitis. Int. J. Oral Maxillofac. Implant..

[20] Renvert S., Lessem J., Dahlén G., Renvert H., Lindahl C. (2008). Mechanical and Repeated Antimicrobial Therapy Using a Local Drug Delivery System in the Treatment of Peri-Implantitis: A Randomized Clinical Trial. J. Periodontol..

[21] Persson G.R., Salvi G.E., Heitz-Mayfield L.J., Lang N.P. (2006). Antimicrobial Therapy Using a Local Drug Delivery System (Arestin) in the Treatment of Peri-Implantitis. I: Microbiological Outcomes. Clin. Oral Implant. Res..

[22] Renvert S., Roos-Jansåker A.M., Claffey N. (2008). Non-Surgical Treatment of Peri-Implant Mucositis and Peri-Implantitis: A Literature Review. J. Clin. Periodontol..

[23] Dörtbudak O., Haas R., Bernhart T., Mailath-Pokorny G. (2001). Lethal Photosensitization for Decontamination of Implant Surfaces in the Treatment of Peri-Implantitis. Clin. Oral Implant. Res..

[24] Hanada N. (2016). The Dental Drug Delivery System for Prevention of Dental Caries. Curr. Oral Health Rep..

[25] Park J.-W., Kook M.-S., Park H.-J., Shet U.-K., Choi C.-H., Hong S.-J., Oh H.-K. (2007). Comparative Study of Removal Effect on Artificial Plaque from RBM Treated Implant. Maxillofac. Plast. Reconstr. Surg..

[26] Cho M.J. (2019). The Effectiveness Focused on Professional Mechanical Tooth Cleaning for Implant Patients Management. Int. J. Clin. Prev. Dent..

[27] Vieira L.F.N., Dias E.C.L.D.C.E.M., Cardoso E.S., Machado S.J., da Silva C.P., Vidigal G.M. (2012). Effectiveness of Implant Surface Decontamination Using a High-Pressure Sodium Bicarbonate Protocol. Implant. Dent..

[28] Augthun M., Tinschert J., Huber A. (1998). In Vitro Studies on the Effect of Cleaning Methods on Different Implant Surfaces. J. Periodontol..

[29] John G., Becker J., Schwarz F. (2016). Effectivity of Airabrasive Powder Based on Glycine and Tricalcium Phosphate in Removal of Initial Biofilm on Titanium and Zirconium Oxide Surfaces in an Ex Vivo Model. Clin. Oral Investig..

[30] Cochis A., Fini M., Carrassi A., Migliario M., Visai L., Rimondini L. (2013). Effect of Air Polishing With Glycine Powder on Titanium Abutment Surfaces. Clin. Oral Implant. Res..

[31] Tastepe C.S., Liu Y., Visscher C.M., Wismeijer D. (2013). Cleaning and Modification of Intraorally Contaminated Titanium Discs with Calcium Phosphate Powder Abrasive Treatment. Clin. Oral Implant. Res..

[32] Subramani K., Jung R.E., Molenberg A., Hammerle C.H. (2009). Biofilm on Dental Implants: A Review of the Literature. Int. J. Oral Maxillofac. Implant..

[33] Amano A., Nakagawa I., Okahashi N., Hamada N. (2004). Variations of Porphyromonas gingivalis Fimbriaein Relation to Microbial Pathogenesis. J. Periodont. Res..

[34] White D.J. (1997). Dental calculus: Recent insights into occurrence, formation, prevention, removal and oral health effects of supragingival and subgingival deposits. Eur. J. Oral Sci..

[35] Rimondini L., Farè S., Brambilla E., Felloni A., Consonni C., Brossa F., Carrassi A. (1997). The Effect of Surface Roughness on Early In Vivo Plaque Colonization on Titanium. J. Periodontol..

[36] Takagi T., Aoki A., Ichinose S., Taniguchi Y., Tachikawa N., Shinoki T., Meinzer W., Sculean A., Izumi Y. (2018). Effective Removal of Calcified Deposits on Microstructured Titanium Fixture Surfaces of Dental Implants with Erbium Lasers. J. Periodontol..

[37] Liu X., Chen S., Tsoi J.K.H., Matinlinna J.P. (2017). Binary Titanium Alloys as Dental Implant Materials—A Review. Regen. Biomater..

[38] Nouri A. (2017). Titanium Foam Scaffolds for Dental Applications. Metallic Foam Bone.

[39] Gehrke S.A., de Lima J.H.C., Rodriguez F., Calvo-Guirado J.L., Aramburú Júnior J., Pérez-Díaz L., Mazón P., Aragoneses J.M., De Aza P.N. (2019). Microgrooves and Microrugosities in Titanium Implant Surfaces: An In Vitro andiIn Vivo Evaluation. Materials.

[40] Dohan Ehrenfest D.M., Coelho P.G., Kang B.S., Sul Y.T., Albrektsson T. (2010). Classification of Osseointegrated Implant Surfaces: Materials, Chemistry and Topography. Trends Biotechnol..

[41] Nicolas-Silvente A.I., Velasco-Ortega E., Ortiz-Garcia I., Monsalve-Guil L., Gil J., Jimenez-Guerra A. (2020). Influence of the Titanium Implant Surface Treatment on the Surface Roughness and Chemical Composition. Materials.

[42] Cervino G., Fiorillo L., Iannello G., Santonocito D., Risitano G., Cicciù M. (2019). Sandblasted and Acid Etched Titanium Dental Implant Surfaces Systematic Review and Confocal Microscopy Evaluation. Materials.

[43] Zhang H., Komasa S., Mashimo C., Sekino T., Okazaki J. (2017). Effect of Ultraviolet Treatment on Bacterial Attachment and Osteogenic Activity to Alkali-Treated Titanium with Nanonetwork Structures. Int. J. Nanomed..

[44] Sahrmann P., Ronay V., Hofer D., Attin T., Jung R.E., Schmidlin P.R. (2015). In Vitro Cleaning Potential of Three Different Implant Debridement Methods. Clin. Oral Implant. Res..

[45] Safi I.N., Hussein B.M.A., Al Shammari A.M., Tawfiq T.A. (2019). Implementation and Characterization of Coating Pure Titanium Dental Implant With Sintered α-TCP by Using Nd: YAG Laser. Saudi Dent. J..

